# Keratinization-like differentiation process forms chitinous dermal sclerites in the hot-vent snail *Ifremeria nautilei*

**DOI:** 10.1098/rspb.2025.1220

**Published:** 2025-08-27

**Authors:** Chong Chen, Satoshi Okada, Hiromi Kayama Watanabe, Katsuyuki Uematsu, Noriyuki Isobe

**Affiliations:** ^1^ X-STAR, Japan Agency for Marine-Earth Science and Technology (JAMSTEC), Yokosuka, Kanagawa 237-0061, Japan; ^2^ Marine Works Japan Ltd., Yokosuka, Kanagawa 237-0063, Japan; ^3^ Biogeochemistry Research Center, Research Institute for Marine Resources Utilization (MRU), Japan Agency for Marine-Earth Science and Technology (JAMSTEC), Yokosuka, Kanagawa 237-0061, Japan; ^4^ Department of Biomaterial Sciences, Graduate School of Agricultural and Life Sciences, The University of Tokyo, Tokyo 113-8657, Japan

**Keywords:** cell differentiation, chitin, convergent evolution, Gastropoda, histology, hydrothermal vent, keratin

## Abstract

Animals produce diverse hard structures for critical functions such as protection, feeding and detoxification. Most animals use the polysaccharide chitin as a framework for this, while vertebrates have switched to using fibrous proteins like collagen and keratin. Vertebrates make structures like skin and horns through a cellular differentiation process called keratinization where cells accumulating keratin die and compact into hard layers—drastically different from chitinous structures, which are secreted directly by living cells. Here, we report remarkable chitinous dermal sclerites that are not secreted but instead produced by a keratinization-like process, in the deep-sea hot-vent snail *Ifremeria nautilei*. These scales bundle to form ‘warts’ on the foot, the framework of which we show to be β-chitin. Microscopic observations reveal that *Ifremeria* scales are not formed by uniform, secreted layers but instead involve cells going through a series of unusual differentiation steps strongly resembling keratinization. The only other gastropod with chitinous dermal sclerites is the phylogenetically distant scaly-foot snail *Chrysomallon squamiferum*, but the scales of *Chrysomallon* form by secretion. Our finding of a chitinous convergence for keratinization opens a new avenue to unveil how such complex terminal cell differentiation processes evolve and may also inspire biomimetic innovation in material sciences.

## 1. Introduction

**Figure 1.**
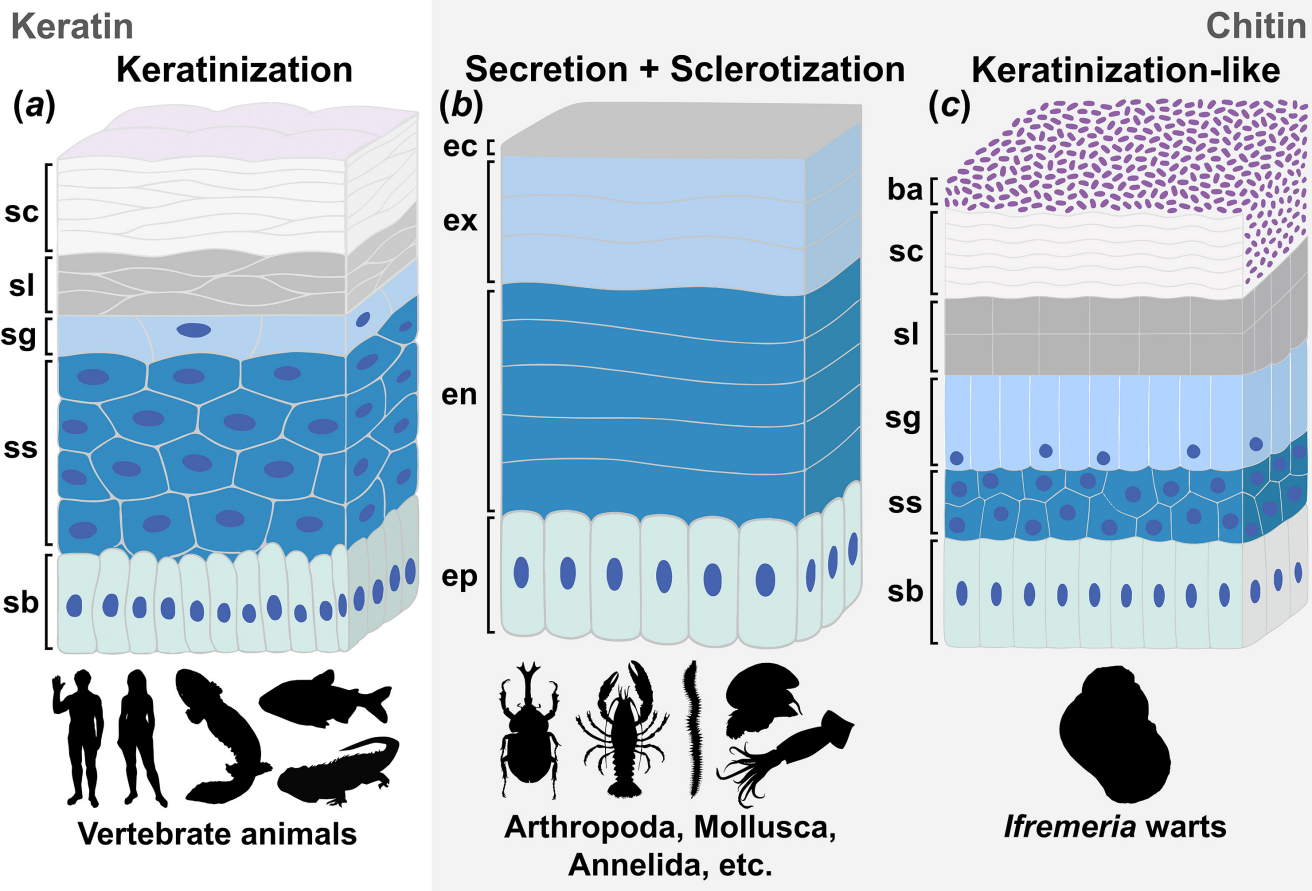
Schematic drawings illustrating the cellular process of animals forming keratinous and chitinous hard parts, dark blue dots inside cells represent the nucleus. (*a*) Keratinization, the typical process of making keratinous hard parts in the integument of vertebrate animals, using human skin as an example (including the stratum lucidum present on thick skins like soles of the feet and palms of the hands). (*b*) Secretion and sclerotization of chitinous hard parts seen in invertebrate phyla such as Arthropoda, Mollusca and Annelida, using insect exoskeleton as an example. (*c*) Keratinization-like formation of chitinous warts newly discovered in *Ifremeria nautilei*. Abbreviations: ba, epibiotic bacteria living inside the cuticle; ec, epicuticle; en, endocuticle; ep, epithelial cells; ex, exocuticle; sb, stratum basale; sc, stratum corneum; sg, stratum granulosum; sl, stratum lucidum; ss, stratum spinosum. Animal silhouettes used are public domain images taken from PhyloPic (https://www.phylopic.org/).

Since the emergence of biomineralization in the Cambrian sparking a rapid diversification of body forms through an evolutionary arms race between predator and prey, hard structures have always been central to the adaptation of animals [1]. Vertebrate animals like fishes and humans use fibrous proteins to make these hard parts, with collagens being the building block for structures like bone and teeth [2], and keratins serving as the basis for components of the integument like skin and nail (figure 1*a*) [3]. However, the polysaccharide chitin is by far the most common organic skeletal component predominantly used by diverse invertebrate animal groups (figure 1*b*) [4]. Chitin comes in two distinct forms: α-chitin with antiparallel packing mostly found in arthropods like insects and crustaceans, and β-chitin with parallel packing occurring in groups like molluscs and annelids [5,6].

Although the detailed formation mechanisms vary among structures, all known chitinous hard parts are formed by chitin molecules secreted extracellularly by living cells. Generally, chitinous integuments are formed by layers of chitin microfibres directly produced by the underlying, usually monolayered, epithelial cells [7]. This soft cuticle is then differentiated through a chemical process known as sclerotization (or ‘tanning’), where proteins are cross-linked with organic compounds and become hardened to confer rigidity. In insect exoskeletons (figure 1*b*), for example, the chitinous procuticle is initially soft and known as the endocuticle, which hardens through sclerotization to form the outer exocuticle; which is in turn covered by a thin proteinaceous and often waxy epicuticle layer [7–9]. In many cases, such as the crab exoskeleton and limpet radula, inorganic components like calcium carbonate and iron oxides are also incorporated, which further enhances the strength of the material [10,11]. The spatial and temporal regulation of this basic formula for chitin secretion and post-secretory modification results in a remarkable diversity of hard-part architectures across many invertebrate phyla.

In contrast, a different process underlies the formation of keratinized vertebrate hard parts such as skin, feathers, hair and horns, where they are not secreted but instead form through a terminal cellular differentiation sequence known as keratinization (figure 1*a*). The vertebrate epidermis exhibits a distinct layering of keratinocyte cells at different stages of producing skin, and other keratinous hard parts are essentially produced by modified versions of this process. These cells originate from a basal cell layer (stratum basale) and then migrate up towards the external surface to form a layer called the stratum spinosum where they stop dividing and instead increase keratin production [12,13]. As keratinocytes differentiate further, they produce more keratin filaments and proteins such as filaggrin that aggregate in a granulose-looking layer known as the stratum granulosum. Then, the nucleus as well as cell organelles are degraded leading to an anucleate, flattened cell as filaggrins are broken down and keratins form cross-links with other proteins, hardening into the final hard, cornified layer (stratum corneum) [14]. In some thickened skin such as the human foot sole and palm, an intermediate clear layer (stratum lucidum) is present between the stratum granulosum and stratum corneum. As such, the keratinous integument of vertebrates comprises of compacted dead keratinocyte cells and there is no extracellular secretion involved.

Deep-sea hydrothermal vents are ‘extreme’ habitats requiring adaptive novelties to conquer successfully, where lush communities of invertebrate animals are powered by microbial energy production through chemosynthesis. The abyssochrysoidean snail *Ifremeria nautilei* is endemic to hot vents in the southwestern Pacific where it often dominates in biomass [15], underpinned by numerous remarkable adaptations. It relies energetically on sulfur-oxidizing bacteria living inside its gill bacteriocyte cells [16,17], meaning it does not need to feed and as a result its gut is greatly reduced [18]. Furthermore, this species also broods a unique free-living pre-veliger larval form with an external cuticle named the ‘Warén’s larva’ [19] in a brooding pouch inside the ventral surface of the foot. This combination of symbiosis, brooding and unusual larval development has made *Ifremeria* a model for studying adaptation in extreme environments. One further striking morphological feature of this species has been much overlooked, however—the numerous ‘warts’ on the side of its foot. These warts have only been briefly mentioned in descriptive literature as positioned around the epipodial furrow, as well as cylindrical in shape and up to 3 mm in length [20–22]. The exact nature of these structures has not been examined, and it remains unclear if they represent a new type of hard part.

Previously, novelties discovered from vent ecosystems included iron-infused, chitinous scales of the peltospirid scaly-foot snail *Chrysomallon squamiferum* [23] with an expected function of detoxifying sulfur waste products of chemosynthetic primary production by its thioautotrophic endosymbionts [24]. This recently evolved feature has been considered the only dermal sclerite among all gastropod molluscs [23,25], but the warts of *Ifremeria* challenges this assumption. Here, we characterize the wart of *Ifremeria* morphologically and biochemically, revealing its nature as chitinous scales—and the first known chitinous hard part to be produced by a cellular differentiation process similar to keratinization. The finding of a novel mechanism of hard part formation in a mollusc also has implications on the interpretation of fossil molluscan sclerites and further highlights the exceptional capacity of lophotrochozoans in evolving new integuments.

## 2. Material and methods

### (a) Sample collection

Individuals of adult *Ifremeria nautilei* Bouchet & Warén, 1991 were collected from an active deep-sea hydrothermal vent at the Vienna Woods field, Manus Basin (03°09.80’S, 150°16.90’W, 2491 m depth) using a suction sampler mounted on the human-occupied vehicle (HOV) *SHINKAI 6500* (Dive #984) during research vessel (R/V) *Yokosuka* cruise YK06-13 Leg 1 (cruise PI: Yohey Suzuki, the University of Tokyo) on 22 September 2006. Once recovered on-board the research vessel, the snails were fixed and preserved in 10% seawater buffered formalin for transmission electron microscopy (TEM), preserved in 80% ethanol for morphological work, or frozen at −80°C for biochemical and physical characterization of the hard parts. The specimens were stored in their original fixation/preservation conditions until further usage in the laboratory. Specimens used have been deposited in the National Museum of Nature and Science, Tsukuba (NSMT), Japan: two specimens in 80% ethanol (NSMT-Mo 79662), two specimens in 10% buffered formalin (NSMT-Mo 79663) and three specimens frozen in a −80°C freezer (NSMT-Mo 79664).

### (b) Rationale and flow of wart characterization

As the warts of *I. nautilei* have not been previously studied in any detail, we combine a number of morphological, biochemical and physical techniques to provide a comprehensive characterization. We initially carried out gross anatomical examination under a light microscope and micro-computed tomography (µCT) in order to reveal the external morphology and distribution of the warts. Then, we produced semi-thin and thin sections of the warts which were examined under light microscopy as well as scanning electron microscopy (SEM) and TEM to characterize the underlying tissue and cellular processes responsible for their formation. Finally, we employed a number of biochemical and physical techniques to identify the building framework of the *Ifremeria* warts. First, we used periodic acid-methenamine silver (PAM) staining [26] and ^13^C cross-polarization (CP) magic-angle spinning (MAS) solid-state nuclear magnetic resonance (NMR) to test whether the warts are formed on a polysaccharide framework (like chitin, as opposed to purely proteinaceous structures like keratinous hard parts). Next, we examined the microscopic transmission Fourier-transform infrared (FT-IR) spectra of the warts to test whether the underlying polysaccharide is chitin. In the last step, we profiled the warts using the wide-angle X-ray diffraction (WAXD) technique with ethylenediamine (EDA) immersion [6] to identify the chitin allomorph used by *Ifremeria* (α-chitin or β-chitin).

### (c) Gross morphology

To investigate the gross morphology of sclerites on *I. nautilei*, one individual preserved in 80% ethanol was dissected under an Olympus SZX7 dissecting microscope after photographs were taken using a digital single reflex camera (Canon EOS 5Ds R with the macro lens EF100mm F2.8L Macro IS USM). Scales were isolated and photographed using the same camera system, a piece of the foot tissue dissected with several warts attached was prepared for μCT scanning (see below).

### (d) Micro-computed tomography scanning and reconstruction

The whole-individual µCT scanning of an adult *Ifremeria* preserved in 80% ethanol has been made available by our previous study [18] and is re-analysed here. Briefly, the animal was decalcified using 0.5 M HCl to remove the shell. Then, any remaining periostracum on the shell was carefully peeled away using tweezers and microscissors. The animal was then gradually rehydrated from 80% ethanol to MilliQ water through a series of 5% incremental steps, each lasting 2 h. This process concluded with two further steps in pure MilliQ water, each also lasting 2 h. The rehydrated specimens were then stained overnight with a 1% iodine solution, followed by immersion in fresh MilliQ water before imaging. Synchrotron μCT was performed at the SPring-8 facility in Hyogo, Japan, using hutch #3 of beamline BL20B2. Scanning was carried out at an energy level of 25 keV using a Hamamatsu Photonics K.K. CCD camera, producing z-stacks comprising 1860 slices with a resolution of 2048 × 2048 pixels. The effective pixel resolution was 13.24 μm. During scanning, specimens were placed in water-filled, X-ray transparent plastic containers and imaged in a hydrated state. Due to its large size, two overlapping scans were conducted and later merged to visualize the entire specimen.

The abovementioned piece of foot tissue with several warts attached from a specimen preserved in 80% ethanol was subjected to μCT scanning. The foot tissue was rehydrated the same way as for the whole-specimen µCT scan as detailed above, and then stained using 1% iodine solution for 24 h before μCT scanning using a ScanXmate-D160TSS105 commercial CT scanner (Comscantecno, Japan) at the Japan Agency for Marine-Earth Science and Technology (JAMSTEC). A total of 1100 projections were obtained, each with a resolution of 908 × 716 pixels with a pixel resolution of 2.60 μm.

**Figure 2.**
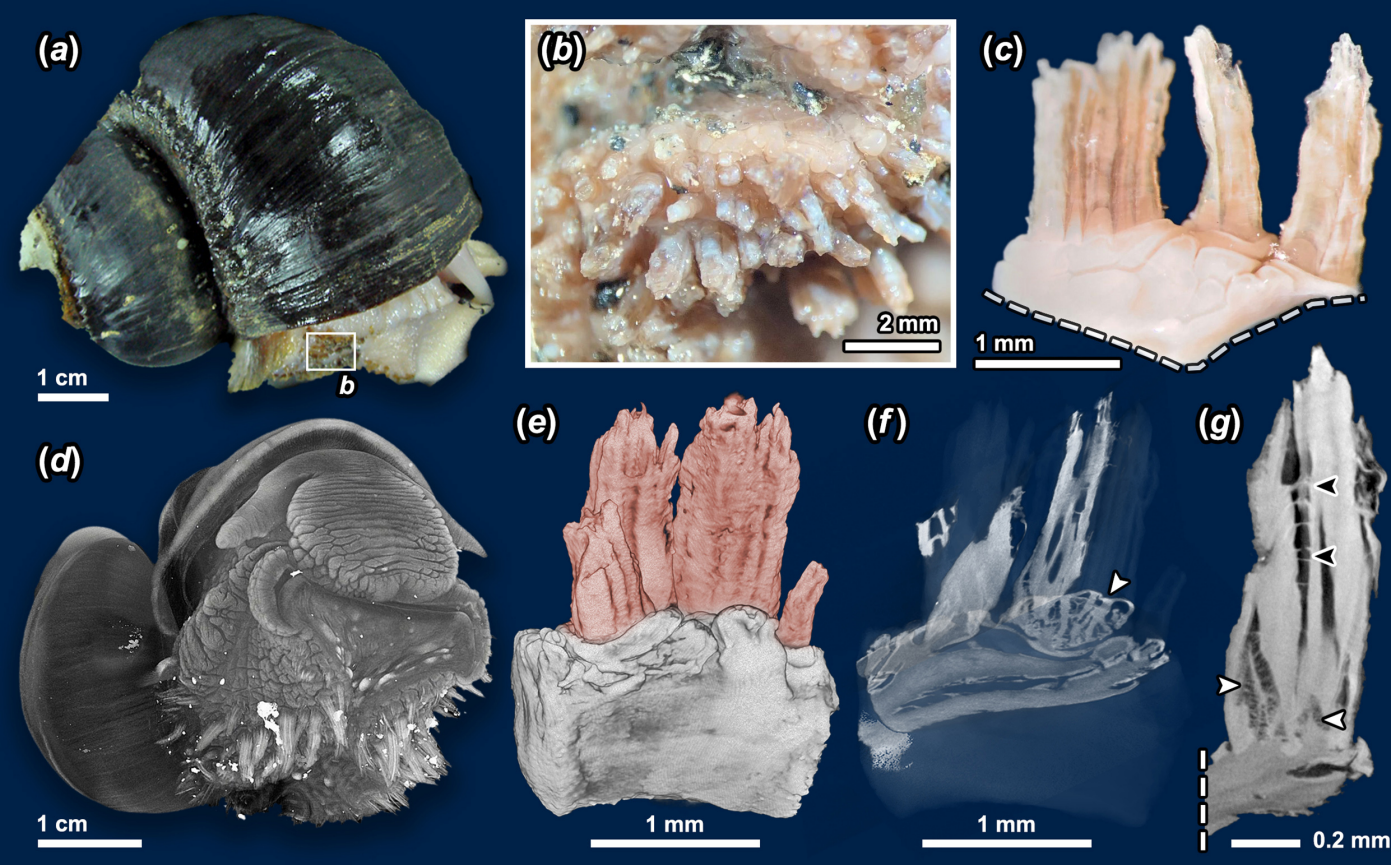
Morphology of *Ifremeria* scales. (*a*) Macrophotography of an adult *I. nautilei* indicating the position of the ‘warts’ on the surface of the foot, enlarged in (*b*). (*c*) Four individual warts isolated and cut away from the foot at the dashed line. (*d*) Synchrotron µCT render of an *I. nautilei* individual, where warts appear brighter indicating denser material with higher X-ray absorbance. (*e*) Surface render of the µCT scan of isolated warts (highlighted in red), with cross-sections from three directions shown in (*f*); white arrowhead indicates the transverse section through the base showing the honeycomb-like structure of the wart where several tube-like scales fuse. (*g*) Sagittal cross-section reconstructed from µCT of a wart with horizontal linkages between scales indicated by black arrowheads and tissue invagination at the base indicated by white arrowheads.

The resulting μCT image stacks were processed in Adobe Photoshop CC, where contrast adjustments and cropping were performed to isolate the region of interest. The image stacks were then imported into Amira v2021.2 (Thermo Fisher Scientific) for surface rendering to produce the images shown in figure 2.

### (e) *En bloc* staining for histology and electron microscopy

For histological investigations and electron microscopy, pieces of the *I. nautilei* foot with warts attached were dissected from two specimens fixed in 10% formalin. Following a rinse in filtered seawater, the warts were cut into slices approximately 0.2−0.4 mm thick using a fresh, sharp razor blade. To optimize preservation of fine ultrastructural details, high-pressure freezing without ice formation was employed, using a Leica EM-PACT2 system [27]. The frozen warts were post-fixed in 2% osmium tetroxide (OsO₄) dissolved in acetone for 3 to 4 days at −80°C. They were then slowly returned to room temperature, and *en bloc* stained with 2% uranyl acetate, which enhances the contrast of the TEM images. After dehydration with acetone, the tissue block was embedded in epoxy resin (TAAB, Aldermaston, UK).

### (f) Histology

The *en bloc* stained and embedded warts were then sectioned at 0.5 μm thickness using a Reichert Ultracut S ultramicrotome (Leica, Wetzlar, Germany) equipped with a 6 mm Histo Jumbo diamond knife (DiATOME, Switzerland). Sections were stained with 0.1% toluidine blue and mounted with Entellan New resin (Merck, Darmstadt, Germany). Light micrographs were acquired using an Olympus BX51 compound microscope. Post-acquisition, images were processed in Adobe Photoshop CC for contrast enhancement.

### (g) Scanning electron microscopy

SEM imaging of the warts was carried out using the same *en bloc* stained wart samples. Energy-dispersive X-ray spectroscopy (EDS) analyses were performed on a Helios G4 UX (Thermo Fisher Scientific) equipped with an Octane Elite Super EDS detector (AMETEK) in JAMSTEC. The SEM–EDS was operated at a landing voltage of 1 kV for SEM imaging and 20 kV for elemental analyses without any conductive coating. A dried, deproteinated wart was mounted on an aluminium pin stub using a piece of double-sided carbon tape (Okenshoji Inc.). The mounted wart was fractured using tweezers to expose its cross-section. When needed, multiple SEM images were stitched together using Adobe Photoshop CC to make a mosaic covering a larger area (see electronic supplementary material, figure S1).

### (h) Transmission electron microscopy

For TEM, the same *en bloc*-stained wart samples were used. Ultrathin sections of the warts at around 70 nm thick were prepared using a Leica EM-UC7 ultramicrotome. These sections were subsequently stained with 2% uranyl acetate and a lead staining solution containing 0.3% lead nitrate and 0.3% lead acetate (Sigma-Aldrich). The TEM observations were conducted at JAMSTEC on a Tecnai G2 20 TEM (FEI) operated at 120 kV.

### (i) Measurements of cell sizes

Cell sizes underlying the warts were measured from light, SEM or TEM micrographs using the image-processing software Fiji [28]. For each measurement, 20 cells (*n* = 20) were measured and their average, standard deviation and range were calculated.

### (j) Periodic acid-methenamine silver staining

Some of the same batch of *en bloc*-stained warts embedded in epoxy resin were sliced into 500 nm semi-thin slices using an ultramicrotome (Ultracut S or EM UC7, Leica) with a diamond knife (45°, Diatome) and collected on a glass slide. The semi-thin sections of warts were PAM stained to visualize polysaccharides [26]. Briefly, a drop of aqueous solution of HIO_4_ (10 mg ml^-1^) was placed on the slide, which was then washed with water after 10 min at room temperature. A drop of thiosemicarbazide aqueous solution (5 mg ml^-1^) was then placed and washed with water after 5 min, followed by a drop of a mixture of methenamine (150 mg ml^-1^), silver nitrate (25 mg ml^-1^), sodium tetraborate decahydrate (25 mg ml^-1^) and gelatine (2 mg ml^-1^) in water and heated at 65°C for 30 min. The glass slide was washed with water, and a drop of tetrachloroauric (III) acid aqueous solution (2 mg ml^-1^) was placed and washed again with water after 5 min at room temperature. The stained semi-thin sections were observed using a Keyence VHX-5000 digital microscope.

### (k) Treatment of warts for biochemical and physical analyses

A total of three *Ifremeria* individuals frozen at −80°C were used for biochemical and physical characterization (^13^C CP/MAS NMR, FT-IR spectroscopic mapping and WAXD). A sharp scalpel was used to harvest the warts from the sides of the foot and then washed in MilliQ water. Then, proteins were removed from cleaned warts using a previously published method [29]. Briefly, in Eppendorf tubes, individual warts were immersed in 7% HCl in an aqueous solution (1 ml) for 6 h at room temperature. The yellowish solution in the tube was removed and washed with deionized water, and 1 ml of 1 M NaOH aq was added. The solution was replaced twice with fresh NaOH aq after 16 and 9 h, respectively. The warts were then washed with water (1 ml), dehydrated in ethanol (1 ml), replaced with *tert*-butyl alcohol (~0.2 ml), frozen at −20°C and freeze-dried to obtain the deproteinated warts, now yellowish in coloration. Optical micrographs were obtained using a Keyence VHX−5000 digital microscope equipped with a VH-ZST zoom lens at each step of the chemical treatment (see electronic supplementary material, figure S2).

### (l) ^13^C cross-polarization magic-angle spinning solid-state nuclear magnetic resonance spectra

The deproteinated warts were used to obtain the ^13^C CP/MAS solid-state NMR spectra through a JEOL JNM-ECAII 500 spectrometer equipped with a 3.2 mm HXMAS probe and ZrO_2_ rotor at 125.77 MHz. The 90° proton decoupler pulse width, contact time, relaxation delay and spinning frequency were 2.5 μs, 2 ms, 5 s and 15 kHz, respectively.

### (m) Fourier-transform infrared spectroscopic mapping

Microscopic transmission FT-IR spectral mapping of the deproteinated warts was conducted using a JASCO FT/IR-6200 type-A spectrometer coupled with an IRT-7000 IR microscope in transmission mode. Deproteinated, *tert-*butanol-dried warts were mounted on a fluorite plate, and spectra were acquired across the 1000−4000 cm⁻¹ range with a spatial resolution of 50 × 50 μm per pixel. Each spectrum was recorded at 4 cm⁻¹ resolution with 256 times of integration.

### (n) Wide-angle X-ray diffraction profiles

Deproteination of the warts was carried out separately from the original frozen material here using 2.5 M aqueous NaOH, followed by thorough washing with water, repeated three times. Purified, deproteinated wart samples were freeze-dried and subjected to a WAXD experiment by a Nano Viewer (Rigaku, Japan) at 40 kV and 40 mA with Cu Kα radiation (*λ* = 1.548 Å).

## 3. Results and discussion

### (a) Morphological characterization

Light microscopy of *I. nautilei* (figure 2*a*–*c*) revealed numerous warts on the side of the foot, densely lined mainly below the epipodial furrow as previously described [20], in transverse rows. These warts were between 1 and 5 mm in length and were darker in coloration compared with the foot musculature. Synchrotron X-ray µCT of a whole individual of *I. nautilei* (figure 2*d*) demonstrated that the warts clearly have different absorption characteristics compared with the foot musculature. Finer-scale µCT of isolated warts (figure 2*e*–*g*) revealed each wart to be lamellar and comprise a bundle of several hollow, tube-like ‘scales’ with occasional bridge-like structures connecting them (figure 2*b,c*). Hereafter, we use ‘wart’ to refer to each bundle, and ‘scale’ to refer to each individual tube-like element in the wart. The base of each wart is honeycomb like, with each scale being invaginated by a soft tissue projection extending from the foot tissue. In the µCT scan, this soft tissue underlying each scale appeared to have a solid, electron-dense core rather distant from the hardened part, with numerous fibre-like extensions from the core that were in contact with the solid part of the scale (figure 2*g*, white arrow).

**Figure 3.**
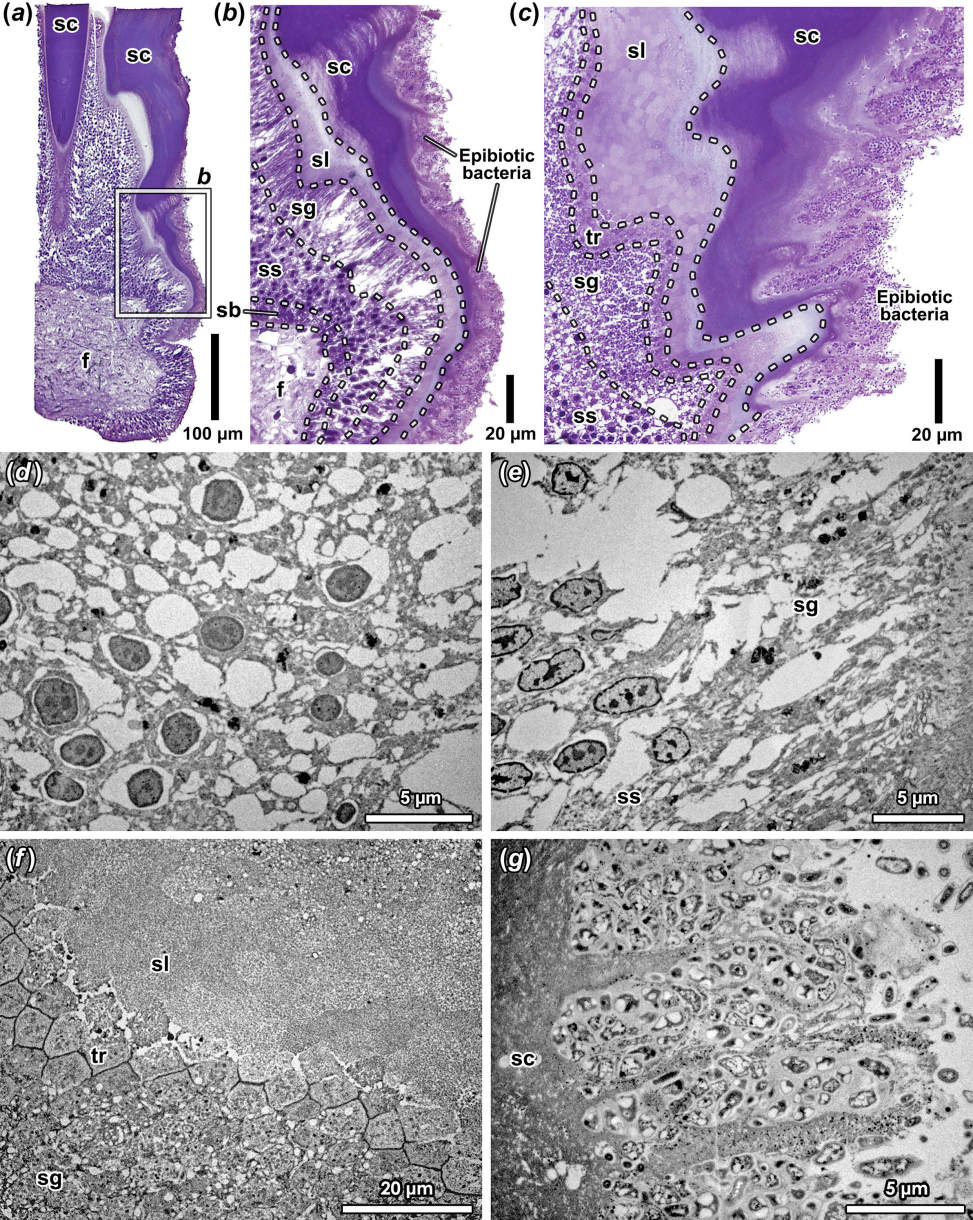
Histology of *Ifremeria* scales. (*a*) Sagittal section through a single scale showing layers of cells between foot tissue and the chitinous scale, enlarged in (*b*) and (*c*) showing different stages of cell differentiation analogous to keratinization [12]. The section is PAM stained. (*d–g*) Transmission electron micrographs of (*d*) the stratum spinosum, (*e*) the stratum granulosum, (*f*) transition from the stratum granulosum to the stratum lucidum and (*g*) epibiotic bacteria densely colonizing the external surface of the stratum corneum. Abbreviations: f, foot tissue; sb, stratum basale; sc, stratum corneum; sg, stratum granulosum; sl, stratum lucidum; ss, stratum spinosum; tr, transition zone between stratum granulosum and stratum lucidum.

Histological sections through the wart (figure 3*a*–*c*) combined with TEM observations (figure 3*d,e*) showed an unusual arrangement of epithelial cells beneath the individual scales. There was not a single layer of columnar epithelial secretory cells as would be typically expected for molluscan chitinous hard parts [30,31], instead there were approximately 8−10 layers of epithelial cells between the foot musculature and the hardened scale. Positionally, it appears that the electron-dense core seen in the µCT scan corresponds to the foot musculature, while the extensions represent the epithelial cells. Different layers of epithelial cells were morphologically distinct, forming three regions. The most basal layer of 1−2 cells thick adjacent to the foot musculature was densely packed with nucleated cells (average length ± s.d. 5.37 ± 0.68 µm, range 4.55−7.24 µm; numbers reported are in the same order hereafter). Above this layer, there was a more loosely packed layer of 4−7 stacked nucleated cells (4.22 ± 0.64 µm, range 3.11−5.63 µm). Then, there is a rather abrupt transition to a layer of much taller, columnar cells (20.41 ± 3.42 µm, range 12.09−25.18 µm). These columnar cells mostly lacked a nucleus, although several nucleated cells were seen these nuclei were always located adjacent to the border with the previous layer where the transition occurred. Observations with TEM (figure 3*e*) unveiled the degradation of electron-dense elements inside the cell when transitioning to the columnar layer, the only equally electron-dense parts of the cell in the previous layer being the nucleus. As such, we interpret that the nucleus is being degraded when transitioning to the columnar layer.

Furthermore, the scale itself was also not uniform in structure. Rather, it was split into a lighter-stained, internal layer comprising numerous polygonal (rectangular to hexagonal) elements (5.39 ± 0.79 µm, range 4.52−7.53 µm) which then transitioned to a darker stained, external layer showing a uniformly grainy texture (figure 3*c*,*g*). From TEM observations of the contact region between the anucleate columnar cells and the internal layer of the scale (figure 3*f*), it became clear that these polygonal elements arose from the columnar cells that become packed into polygonal elements at the transition zone. At the transition zone, the borderline between each polygonal element is still clearly seen, but soon they fuse together to form the internal layer of the scale, with only remnants of the polygonal outline still visible. Moving towards the outer surface of the scale, these polygonal outlines become less and less clear, likely due to compaction, and finally they become completely homogenous and form the outer layer of the scale. In SEM and TEM observations, there was no evidence of striations in longitudinal sections of the outermost layer. The outer surface of the scale is densely packed with bacterial cells (figure 3*g*), many also infiltrating into the cuticular part of the scale.

### (b) Scale-formation process

The scale-formation process of *Ifremeria* observed is summarized in a schematic shown in figure 1*c*. As described above, the *Ifremeria* scales are formed by dense packing of enucleated cells, which ultimately become polygonal building blocks that appear to be compacted to form the hard scale. This is distinctly different from the typical secretion process of invertebrate chitinous integument [7,9,31] where there would be growth lines of chitin-rich secretions above a uniformly structured epithelium, with new layers of secretions pushing the old ones outwards and undergoing sclerotization (figure 1*b*). Instead, the observed process is strongly reminiscent of the formation of hard structures through cellular differentiation in keratinization and cornification [32] of vertebrate animals (figure 1*a*). In mammalian skin, for example, keratinocytes first proliferate in the stratum basale and then move up to form several layers in the stratum spinosum that synthesize keratin filaments [33]. As these cells move further suprabasally, they shed the nucleus and organelles in the stratum granulosum. Finally, the dead cells are compacted like bricks [13] in the stratum lucidum (only found in thickened skin) and stratum corneum (common across all keratinous structures) to form the hard structure.

We can find layers analogous to all these steps of keratinization in the scale-producing epidermis of *Ifremeria* (figure 3*b*–*g*), and therefore we adopt the same naming scheme [12] for cell layers seen in the keratinization-like process of *Ifremeria*. The most basal layer is therefore called the stratum basale, the several layers of nucleated cells above it form the stratum spinosum and the enucleated columnar cells represent the stratum granulosum. Our observations indicate the cells in the stratum spinosum likely originate by cell division in the cells below in the stratum basale, similar to mammalian skin. The apparently intracellular degradation of the nucleus in the stratum granulosum is also remarkably similar to the process in vertebrate keratinization [12,33], although the formalin fixation of our limited specimens was too poor for us to observe the potential degradation of organelles (figure 3*e*). One important difference between the *Ifremeria* scale and mammalian epidermis is that the cell sizes in the stratum spinosum are much smaller than typical mammalian keratinocytes (average 4.22 µm versus 10−15 µm in human keratinocytes [13]). Another key difference is that the cells in the stratum granulosum of *Ifremeria* are stretched vertically into columnar structures instead of flattened out like mammalian skin [33].

In the hardened part of the scale, the inner part where the polygonal elements are still visible is analogous to the stratum lucidum, and the outer uniform layer is likened to the stratum corneum. The stratum lucidum of *Ifremeria* is similar to that of vertebrates in that the dead cells are clearly visible in a more translucent layer than the stratum corneum [12], but the cells are not as flattened yet, being still polygonal in outline. Conversely, the stratum corneum of *Ifremeria* is distinct from the vertebrate equivalent in that the polygonal elements are no longer visible under SEM or TEM; although sometimes layering is seen under light microscopy (figure 3*b*). In vertebrate stratum corneum, typically the dead cells are still visible as flattened, scale-like elements that make up the skin like bricks, which are stuck together by lipids [13]. A transition zone between the stratum granulosum and stratum lucidum/corneum is also present in the *Ifremeria* scales (figure 3*c*,*f*) like in the vertebrate skin [12]. The *Ifremeria* stratum corneum sometimes exhibits several zones of different density and coloration (electronic supplementary material, figure S1), likely indicating different levels of compaction. The external surface of the stratum corneum in *Ifremeria* exhibits numerous irregular projections (figure 3*c*), which may eventually be lost in a way similar to the outermost layer of keratinous hard parts shedding away (desquamation) [33].

### (c) Identification of the building framework

**Figure 4.**
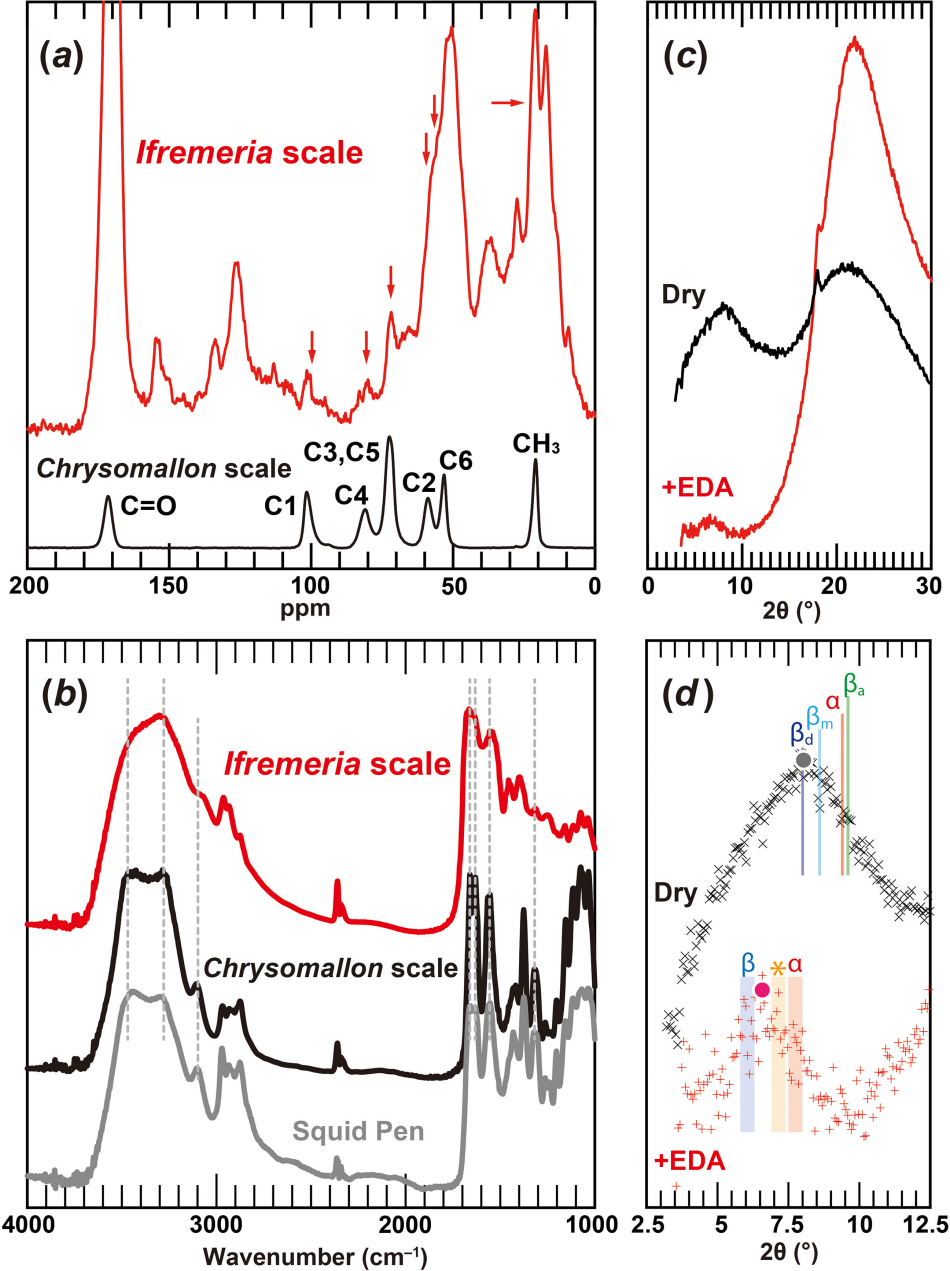
Characterization of chitin in *Ifremeria* scales. (*a*) ^13^C CP/MAS NMR spectra of native *Ifremeria* scales and chitin obtained from previously characterized *Chrysomallon squamiferum* scales [29]. (*b*) FT-IR spectra of purified *Ifremeria* scales compared with *C. squamiferum* scales and squid pen from *Todarodes pacificus* previously characterized in [29]. (*c,d*) X-ray diffraction profiles of (*c*) dry, de-protenized *Ifremeria* scales and EDA-soaked, de-protenized *Ifremeria* scales; (*d*) is a higher magnification of (*c*). The labels α, β_a_, β_m_ and β_d_ represent the peak positions of α-chitin, anhydrous β-chitin, β-chitin monohydrate and β-chitin dihydrate from the literature data, respectively. Coloured dots represent the peak top positions of these profiles. Shades denoted as α and β are the reported peak position of the EDA complex of α- and β-chitin [6] with a margin of potential experimental error of plus or minus 2*θ* = 0.25°. The shade marked with an asterisk is the tentative peak position of the EDA/chitosan complex, with a margin of potential experimental error of plus or minus 2*θ* = 0.25° to the peak position observed in the literature. Coloured dots represent the peak top position of these profiles.

The possible presence of polysaccharides forming the framework of *Ifremeria* scales was detected in PAM staining (electronic supplementary material, figure S3). We then subjected *Ifremeria* scales to solid-state ^13^C CP/MAS NMR spectra analysis (figure 4*a*) in order to identify its chemical structure. The spectrum showed sharp peaks originating from peptides, but all signals characteristic to polysaccharides were also detected and indicate the presence of a low amount of polysaccharides.

The FT-IR spectra (figure 4*b*) displayed absorbance bands at 3452, 3286, 3092, 1661, 1625 and 1549 cm^−1^, corresponding to O–H stretching, N–H stretching (asymmetric), N–H stretching (symmetric), amide I (singly H-bond), amide I (doubly H-bond) and amide II, respectively [34] which are characteristic of chitinous samples, as also seen in purified squid pen and *Chrysomallon* scales. This clearly reveals the organic framework of *Ifremeria* scales as chitin. The broad spectrum indicates that the chitinous components are poorly crystalline. In addition, the peak at 1310−1320 cm^−1^ characteristic of an *N*-acetylglucosamine unit [35] is not evident, indicating the chitin is highly de-acetylated and at an intermediate state between chitin and chitosan, the de-acetylated form of chitin.

The WAXD profile (figure 4*c*) of dried and deprotenized *Ifremeria* scales (electronic supplementary material, figure S2) showed broad peaks generally corresponding to dihydrate β-chitin. Using the EDA immersion technique, which can identify chitin allomorphs based on peak position, even in samples with small crystal sizes [6], we found the peak position of EDA-complexed *Ifremeria* scales was located in-between the known positions of β-chitin and chitosan (figure 4*d*). These collectively suggest that the chitin in the *Ifremeria* scale is β-chitin like other molluscan chitinous structures [6,36], but is poorly crystalline. Therefore, despite having a keratinization-like underlying cellular process for its making, the *Ifremeria* wart is indeed a chitinous structure.

### (d) Potential functions of the scales

*Ifremeria nautilei* is only the second living gastropod species known to produce dermal sclerites, after *C. squamiferum* [37]. Both species live in deep-sea hydrothermal vents and rely on intracellular sulfur-oxidizing bacteria for nutrition [17,38]; in *Chrysomallon* the scales are not for protection but rather act as sites of sulfur detoxification by active secretion of sulfur into the scales by the snail [38]. The presence of analogous scales in two vent snails with similar ecology suggests similar function, but our EDS analysis of the scale (electronic supplementary material, figure S4) did not reveal high sulfur content or enrichment in metals. Both the total count per second and Cl signals (representing signals from the epoxy resin used for embedding) in our EDS spectra indicate the outer corneous layers are denser, and therefore with less resin infiltration—in line with a gradual cornification process similar to keratinization.

Taken together with the different formation processes and the fact that *Ifremeria* is phylogenetically distant from *Chrysomallon* at the subclass level [39], our results indicate these two scales have independent evolutionary origins and probably different functions. Although the exact function remains unclear, the scales could potentially protect *Ifremeria* snails against predators such as bythograeid crabs that are common in the same habitat [15], especially if the foot cannot be retracted when brooding the Warén’s larvae in the brooding chamber on the ventral side of the foot [19]. The external surface of the scales is covered by dense layers of epibiotic bacteria which are often also embedded inside the stratum corneum (figure 3*c*,*g*). As episymbionts are known to play important ecological functions in many hot-vent animals [40–42], we hypothesize that the scales of *Ifremeria* may be important sites for hosting specific epibiotic bacteria. The most similar example is perhaps the vent aplacophoran genus *Helicoradomenia,* which hosts epi- and endocuticular symbionts, although it is unclear whether they play any nutritional role for the host [43]. The Pompeii worm *Alvinella pompejana* has special epithelial projections associated with a dense bacterial community that has been suggested to act as thermal insulation from the hot-vent fluid and/or help the host animal detoxify chemicals in its ‘extreme’ microenvironment [44,45]. *Ifremeria* scales may have similar functions, offering distinct ecological advantages in its unusual habitat.

### (e) Implications of *Ifremeria* scales

Our discovery of the first chitinous hard part made by cell terminal differentiation highlights the capacity of molluscs, and lophotrochozoan animals in general, to rapidly evolve novel hard parts. Lophotrochozoan animals like snails have a highly conserved ‘biomineralization toolkit’ that can be traced at least to the early Cambrian [46], a deep homology that allows them to reshuffle these toolkit genes to rapidly evolve a diverse range of hard structures [25]. The *Ifremeria* warts formed by bundles of several hollow scales interconnected by occasional bridges expands the known range of morphology for molluscan hard parts but is clearly recently evolved, since the genus is thought to have originated around the early Cretaceous and no other member of its subclass exhibits dermal scales [47]. In lophotrochozoans, bundles of chitinous elements with tubular internal canals such as annelid chaetae [48] and the Kölliker’s organ in octopuses [49] are typically secreted by epithelial cells densely covered in microvilli [50]. Although each microvilli secreting individual elements is minute (down to ~50 nm), the secretion and sclerotization process is essentially similar to typical chitinous structures and possesses a striated microstructure in longitudinal views [50].

Molluscs boast an exceptional diversity of hard parts in the fossil record, with different types of sclerites often used to support the systematic placement of extinct lineages such as the similarities between the sclerites of Cambrian fossils such as *Sinosachites*, *Halkieria* and *Wiwaxia*, and those of living aculiferan molluscs including aplacophorans and chitons [50,51]. The *Ifremeria* warts are hollow and lamellar sclerite-like features arranged in transverse rows on the foot—arguably similar to the arrangement of solid chitinous sclerites of *Wiwaxia* organized in loose fans across transverse rows, for which there have been no satisfactory living comparables [52]. *Ifremeria* warts have no signature of microvillar secretion and are clearly not homologous to *Wiwaxia* sclerites, but it reminds us that similar structural outcomes can be produced convergently through entirely different developmental routes [31]. The complex internal structure of the *Ifremeria* wart produced in a lineage otherwise lacking a sclerotome through a totally unexpected process further cautions against the use of superficial resemblance in small hard parts as evidence for homology in the interpretation of early molluscan fossils [31].

The keratinization-like process in *Ifremeria* scales parallels vertebrate epidermal differentiation in structures such as reptilian scales and mammalian nails [32], but with chitin instead of keratin—making *Ifremeria* a unique model to study this chitin biosynthesis process. Since β-chitin only comprises a small part of *Ifremeria* scales, it likely serves as a framework for structural proteins like other chitinous structures [53]. Future research on the molecular, cellular and ecological underpinnings of this chitinous formation process could reveal how and why animals across different phyla independently converged on similar strategies, using fundamentally different biochemical constituents. Additionally, the study of *Ifremeria* scales may inform the field of biomimetics. Chitinous structures like limpet teeth are the strongest known biomaterial [10], and understanding the new way *Ifremeria* makes chitin-protein hard parts could inspire the engineering of novel chitin- and chitosan-based biocomposites [54] with potential applications such as protective coatings or soft robotics—especially useful as chitin is biodegradable.

Our results reveal a striking case of evolutionary convergence in analogous terminal cell differentiation across distant animal lineages, challenging long-held distinctions between hard structure formation processes between invertebrates and vertebrates that use polysaccharides and proteins as scaffolds, respectively [3,4]. At the moment our findings are limited by the availability of deep-sea material and that *Ifremeria* cannot be reared alive, but future research—particularly those requiring fresh or live specimens—will likely open new frontiers in understanding the deep evolutionary plasticity of epithelial differentiation programmes across the animal kingdom.

## Data Availability

Image stacks from μCT scans are available in Figshare; the whole *Ifremeria* adult individual at [18,55] and the isolated warts/scales at [56]. All other relevant data are contained within this article. Supplementary material is available online [57].
